# Mechanical Characterization of Cardanol Bio-Based Epoxy Resin Blends: Effect of Different Bio-Contents

**DOI:** 10.3390/polym17030296

**Published:** 2025-01-23

**Authors:** Andrea Iadarola, Pietro Di Matteo, Raffaele Ciardiello, Francesco Gazza, Vito Guido Lambertini, Valentina Brunella, Davide Salvatore Paolino

**Affiliations:** 1 Department of Mechanical and Aerospace Engineering, Politecnico di Torino, Corso Duca degli Abruzzi 24, 10029 Turin, Italy; davide.paolino@polito.it; 2 Department of Chemistry, Università degli Studi di Torino, Via Pietro Giuria 7, 10125 Turin, Italy; pietro.dimatteo@unito.it (P.D.M.); valentina.brunella@unito.it (V.B.); 3 Material Technical Expertise Department, Centro Ricerche Fiat (Stellantis), Corso Giovanni Agnelli 220, 10135 Turin, Italy; francesco.gazza@crf.it (F.G.); vitoguido.lambertini@crf.it (V.G.L.)

**Keywords:** cashew nutshell liquid, carbon footprint, composites, automotive

## Abstract

This study investigates the impact of an increased bio-content on the mechanical properties of bio-based epoxy resins. Cardanol-based epoxy and novolac resins (65% and 84% bio-content, respectively) were combined with two commercial cardanol-based epoxy systems to achieve higher total bio-contents. Quasi-static tensile tests showed that resin blends with up to 40% bio-content maintain tensile properties comparable to traditional formulations, with a glass transition temperature (T_g_) suitable for automotive requirements. The results highlight that an increased bio-content enhances flexibility and viscoelastic behavior. Additionally, the tests showed that epoxy resins with a high bio-content represent a sustainable alternative with reduced environmental impact. This work benchmarks novel cardanol-based epoxy formulations with existing bio-based systems, supporting their industrial application.

## 1. Introduction

### 1.1. Conventional Epoxy Systems and Environmental Issues

In recent decades, epoxy resins have found a wide range of applications in many industrial sectors, especially in the aerospace and automotive industries, where the use of epoxy matrix composite materials has intensified due to their light weight and mechanical properties [1,2]. The most-used petrol-based epoxy resins are derived from the diglycidyl ether of bisphenol-A (DGEBA), which is synthesized through the reaction of bisphenol-A (BPA) and epichlorohydrin (ECH) [2,3]. At present, the production of these petroleum-based reactants still generates a high quantity of CO_2_ [4], and they have energy-intensive manufacturing processes that do not allow for a reduction in the carbon footprint. Given the environmental and economic concerns related to petrochemical resources and European regulations [5] aiming to lower the overall carbon footprint in automotive production, the use of green polymers and bio-composites represents one of the most promising available solutions to reduce the carbon footprint of components produced with these resins [6].

### 1.2. Bio-Based Epoxy Systems

In recent years, the use of bio-based epoxy systems in the production of composite materials has increased due to the possibility of reducing the impact of CO_2_ (on average, 40% carbon footprint reduction [7]) and having comparable mechanical properties to common petrol-based epoxy systems [6]. In order to make DGEBA epoxy resins more sustainable, several sources of bio-based aromatic molecules have been suggested [3] to replace BPA, including cardanol, vanillin, rosin, lignin, tannins, and limonene [8]. Moreover, DGEBA epoxy resins can be substituted by novolac epoxy resins that are synthesized by reacting phenolic novolac resin with epichlorohydrin instead of BPA. Also, ECH can be produced using renewable resources; for example, Epicerol^®^ is a manufacturing process (developed and patented by Solvay) that is based on the use of natural and renewable glycerol (from the bio-diesel industry) as a precursor in ECH production [9] instead of petrol-derived propylene. Thanks to a similar synthesis process (GreenPoxy^®^ process [10]), Sicomin was able to reduce carbon dioxide emissions by 60% [11]. According to data obtained by the company [10], the carbon footprint (climate change—GWP100) of their bio-based systems is, on average, 40% less than a common petroleum-based system. Among these approaches, the most interesting is the one based on using cardanol [12,13,14]. Cardanol is a phenolic lipid obtained by cashew nutshell liquid (CNSL), a natural, non-food chain and annually renewable biomaterial extracted from a soft honeycomb structure inside cashew nutshells [13,14]. The epoxidation of cardanol can be accomplished by using ECH in the presence of caustic soda as a catalyst [14]. CNSL can be decarboxylated and distilled to yield high-purity cardanol, a highly desirable alkyl phenolic compound in the chemical industry [12,13,14]. The distillation residue can produce resilient friction particles and binders for the automotive brake lining industry [15]. Moreover, CNSL-based resins can substitute petroleum-based phenolic resins as rubber modifiers to improve the carbon footprint and lower the toxicity of the final compound [16]. Cardanol-based resins, which contain a certain amount of CNSL with a long aliphatic structure, can provide flexibility and resilience to the material to enhance molecular mobility within the cured samples [17].

### 1.3. Literature Review

In the literature [17,18,19,20,21,22,23], some commercial and non-commercial cardanol-based epoxy resin systems have already been studied. Usually, they exhibit lower T_g_ [8] and lower mechanical properties [8,18] compared to conventional petrol-based epoxy resin systems or commercially available bio-based epoxy systems. Despite these studies, no unique study has investigated the mechanical properties of epoxy resin with an increased bio-content and the relative variation in T_g_. In 2008, Unnikrishnan and Thachil [18] distilled cardanol from CNSL to produce new cardanol-based epoxy compounds blended with commercial DGEBA resins. The introduction of 20 mol% cardanol into BPA resulted in a resin having reduced tensile, impact, and compressive strengths upon curing by a polyamine hardener. An increase in the cardanol content led to a decrease in the tensile and compressive strengths and a sharp increase in elongation at break. In 2014, Jaillet et al. [19] formulated epoxy materials using a cardanol-based epoxy resin (Cardolite NC-514) and two commercial amines as hardeners: isophorone diamine (IPDA) and Jeffamine D400. They observed that the structure of NC-514 is characterized by a long aliphatic chain, which increases the distance between the epoxy groups, reducing the cross-linking density compared to DGEBA resins, which affect the mechanical properties. They determined a ratio of 70% of the epoxy groups and 30% of the opened epoxy ring in NC-514. Moreover, they performed dynamic mechanical analyses (DMA) on NC-514/IPDA and DGEBA/IPDA formulations, obtaining similar storage modulus values. By contrast, the T_g_ value was significantly lower for the formulation modified with the cardanol-based resin. Gour et al. [21] investigated the possibility of toughening a non-bio-based epoxy novolac resin, poly[(phenylglycidyl ether)-co-formaldehyde] (PPGEF), through the addition of cardanol bio-based epoxy resins (Cardolite NC-514 and Cardolite NC-547) in different weight amounts. They observed a decrement in the T_g_ values by increasing the cardanol-based resin content. Moreover, they observed [20] an increment in their flexibility and a decrement of about 10% and 12.5% in tensile strength and elongation, respectively, by adding 30% in weight of NC-547. By contrast, tensile strength and elongation showed an increment of about 6.5% and 10.5%, respectively, by adding 30% in weight of NC-514. In 2021, Mu and Vaughan [17] studied the glass transition temperature of cardanol-based epoxy systems. Differential scanning calorimetry (DSC) analysis demonstrated that the T_g_ values of the investigated epoxy systems ranged from 67 °C to 122 °C. The epoxy systems characterized by a lower bio-content showed higher T_g_ values. In a previous work of the authors [22], in 2023, a cardanol-based epoxy system (27% bio-content) was blended with a cardanol-based epoxy novolac resin (84% bio-content). In particular, the high-bio-content epoxy novolac resin (Cardolite NC-547) was mixed with the cardanol-based epoxy system (Cardolite^®^ FormuLITE™ 2502A + 2401B) in different weight loadings to obtain resin mixtures characterized by a total bio-content higher than 27%. Tensile tests on four resin blends characterized by different total bio-contents (27%, 31%, 41%, and 51%, calculated on biomass used in production) were carried out considering three different strain rates. Quasi-static (0.1 mm/s) and dynamic (10 mm/s and 100 mm/s) tensile tests shown that the increase in bio-content led to lower Young’s modulus and ultimate strengths, decreasing with a linear trend. In particular, the highest bio-content blend showed a total reduction of about 62% and 67% in modulus and strength, respectively. The DSC analysis showed a decrease in T_g_ values by increasing the content of NC-547, providing flexibility to the final epoxy resin blend, also confirmed by the decrease in modulus values. The present paper aims to study the effect of the increase in the mechanical properties of the bio-content in epoxy-based resin systems to present a ready-to-use alternative for reducing the carbon footprint of composite materials. Two high-bio-content cardanol-based epoxy and novolac resins (65% and 84% bio-content, respectively) were blended with two commercial epoxy systems, containing 34% and 45% bio-content, to achieve a higher overall bio-content in the final epoxy resin blend. The highest bio-contents analyzed in this work were 50% and 58%, respectively, for the two neat epoxy systems. Since the increase in bio-based content also raises the material viscosity, the maximum percentage was selected so as to achieve a resin that would remain pourable for the fabrication of the specimens and, thus, could also be used in fabrication processes such as resin infusion and compression molding. Quasi-static (0.1 mm/s) tensile tests were carried out to assess the tensile behavior of the different resin blends at increasing bio-contents. The effect of the bio-content increment on the T_g_ values was investigated by performing DSC analyses on all bio-based resin blends. This work allowed us to compare the mechanical properties of the cardanol-based epoxy resins, starting from the same neat formulation and obtaining resin blends with increased bio-content, offering a benchmark for what is available in the literature and on the market for the production of bio-based composites for industrial applications, in particular for the automotive industry.

## 2. Materials and Methods

### 2.1. Bio-Based Epoxy Blends

The epoxy resins and hardeners used in this study were purchased from Cardolite Specialty Chemicals Europe N.V. (Mariakerke, Gent, Belgium). The epoxy resins were Cardolite^®^ FormuLITE™ 2501A, Cardolite^®^ NC-514, and Cardolite^®^ NC-547. Cardolite 2501A is based on DGEBA with cardanol as a precursor. Resin 2501A is characterized by a bio-content of about 34% and an epoxy equivalent weight (EEW) of 200 g/eq (the weight of resin in grams which contains one gram equivalent of epoxy groups [24]). Cardolite NC-514 is a cardanol-based di-functional glycidyl ether epoxy resin that, unlike other flexible resins, exhibits the reactivity and chemical characteristics of a traditional BPA resin. The chain flexibility resulting from the 8 carbons that separate the aromatic groups increases the flexibility when mixed with traditional resins without losing its chemical and mechanical properties [25]. NC-514 resin is characterized by a bio-content of about 65% and an EEW equal to 425 g/eq. Moreover, a cardanol-based epoxy novolac resin (Cardolite^®^ NC-547) was also used. NC-547 resin is a poly-glycidyl ether of an alkenyl phenol-formaldehyde novolac resin characterized by a very high bio-content (84% biomass) and an EEW equal to 840 g/eq. The reactive epoxide groups and the long alkenyl side chains impart flexibility in standard cured epoxy resin systems [26].

The hardeners used were Cardolite^®^ FormuLITE™ 2401B and 2002B, both amine-based hardeners characterized by a bio-content of about 33% and 67%, respectively. The amine hydrogen equivalent weight (AHEW) of hardener 2401B is 62 g/eq., whereas the AHEW of hardener 2002B is 104 g/eq. The AHEW is the weight of the hardener in grams, which contains 1 g of amine hydrogen equivalent to active hydrogen groups [24]. The manufacturer states that all these components contain both fossil and natural compounds derived from cardanol (bio-content calculated on the biomass used during production).

The supplier suggests the use of two commercial epoxy systems composed of part A (epoxy resin) and part B (ammine hardener). Table 1 reports the chemical and mechanical properties of the two cardanol-based epoxy systems (2501A + 2401B and 2501A + 2002B) used as starting points for blending (neat epoxy systems).

All data reported in this section were provided by the supplier of the resin [25,26,27,28]. The neat cardanol-based epoxy systems (Table 1) were mixed with NC-514 and NC-547 resins in different weight loadings (before curing) to obtain different blends characterized by higher total bio-contents than the respective neat epoxy systems. For the sake of clarity, the neat epoxy systems 2501A + 2002B and 2501A + 2401B were called M1 and M2, respectively.

Blending different resins and hardeners, characterized by different EEW and AHEW, requires the redefinition of the ratio of stochiometric parts by weight of resin and hardener. The evaluation of the EEW of the resulting resin blends was necessary to calculate the stochiometric parts by weight of curing agent per 100 parts of epoxy resin (PHR) [29,30], as shown by Equations (1) and (2) [30].

(1) $$EEW_{Epoxy\;Resin\;Blend}=\frac{Total\;weight\;of\;epoxy\;resin\;blend}{\frac{Weight_{A}}{EEW_{A}}+\frac{Weight_{B}}{EEW_{B}}}$$

(2) $$PHR=\frac{AHEW_{Curing\;Agent}}{EEW_{Resin\;Blend}}\times 100$$

The total bio-content of each new epoxy blend was computed using the rule of mixture (Equation (3)), starting from the bio-content of each component provided by the supplier.

(3) $$\frac{{(m}_{Resin\;A}\times \%bio_{Resin\;A})+{(m}_{Resin\;B}\times \%bio_{Resin\;B})+{(m}_{Hardener}\times \%bio_{Hardener})}{m_{Resin\;A}+m_{Resin\;B}+m_{Hardener}}$$

where $m_{Resin\;A},m_{Resin\;B},and\;m_{Hardener}$ and $\%bio_{Resin\;A},\%bio_{Resin\;B},and\;\%bio_{Hardener}$ are the weights and bio-contents of resin 2501A (Resin A), resin NC-547 or NC-514 (Resin B), and hardener 2002B or 2401B, respectively.

The values obtained from these calculations are reported in Table 2 and Table 3 for all the blends produced using the M1 and M2 epoxy systems, respectively.

The material contents shown in Table 2 and Table 3 are intended as representative calculations made on 100 g of resin blend, considering the increasing weight of the NC-547 (or NC-514) resin. Since we wanted to limit the viscosity of the resin to ensure that it would be pourable to allow manufacturing, a pourability analysis was performed. Therefore, the quantities were decided starting from the resin with the highest bio percentage, and then intermediate values were defined. The nomenclature used for this work presents direct information that is useful for correctly identifying the epoxy blends. The first term indicates the neat epoxy resin system used (M1 or M2). The second term (NEAT, N or C) indicates whether the blend was produced as received (NEAT) or using the epoxy novolac resin NC-547 (N) or the cardanol epoxy resin NC-514 (C). The last term refers to the total bio-content of the obtained epoxy blend, rounded up. A two-step mixing process was used to prepare the epoxy resin blends. Initially, the 2501A resin was mixed with NC-547 (or NC-514) until a completely homogeneous mixture was obtained, considering the different weight loadings calculated in Table 2 and Table 3. Subsequentially, the hardener was added in stoichiometric amounts (Table 2 and Table 3) for the 2002B and 2401B ammine hardeners, respectively. Finally, the epoxy formulation (resins + hardener) was thoroughly mixed at ambient temperature for 10 min, and a degassing was performed (10 min) to remove any entrapped air bubbles. Both mixing processes were carried out until the achievement of a completely homogeneous mixture.

### 2.2. Specimen Manufacturing

**Figure 1:**
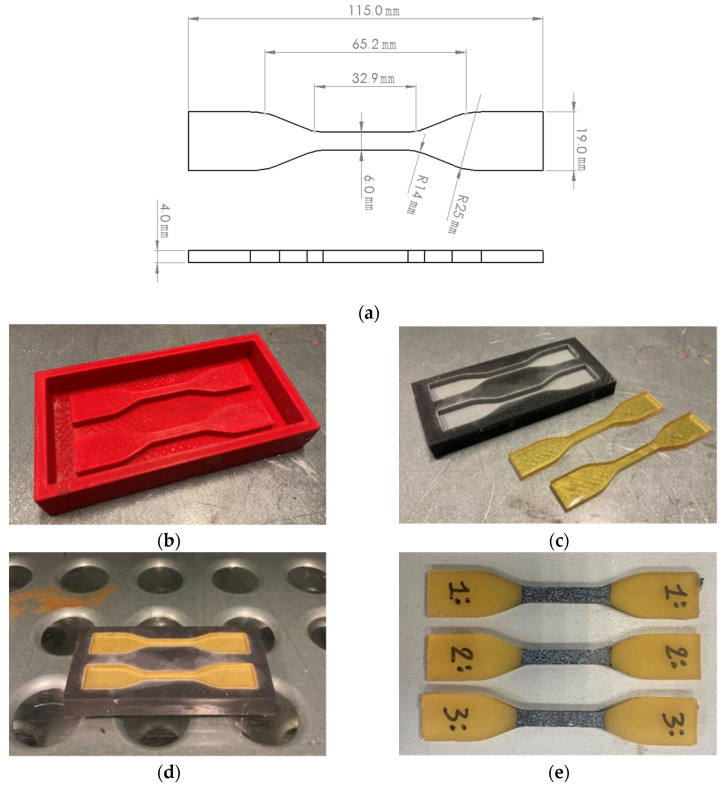
Specimen production: (**a**) specimen geometry [31]; (**b**) negative mold; (**c**) silicon mold; (**d**) silicon mold in the curing oven with liquid resin; and (**e**) smoothed specimen with a speckle.

The geometry of the specimen (Figure 1a) adopted for testing the resin blends followed the specifications of the standard ASTM D638 [31].

**Figure 2:**
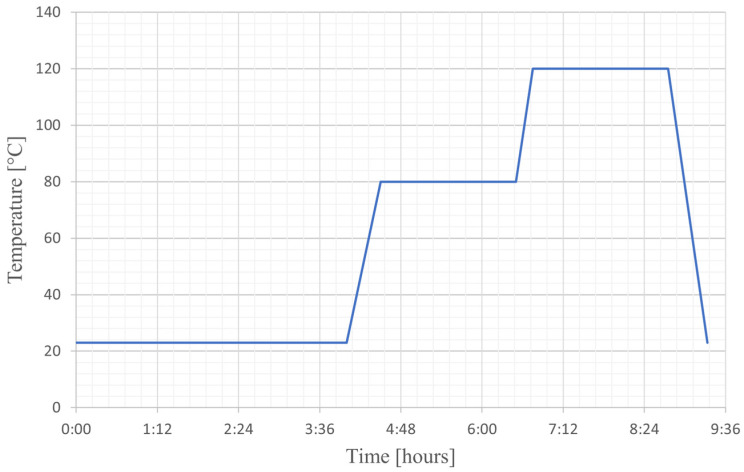
Curing ramp.

A negative mold was first produced (Figure 1b) using a 3D printer for manufacturing the specimens. A two-part condensation cure silicone rubber (CS25 condensation cure silicone rubber, Easy Composites, Stoke on Trent, UK) was poured into the negative mold. After curing for 24 h at room temperature, the silicon mold with the specimen geometry was obtained (Figure 1c). This mold was used to contain the liquid resin blend during the curing stage (Figure 1d). Before casting the resin mixture into the silicon mold, the degassing phase was performed to avoid the presence of bubbles and, therefore, voids in the final material. Subsequently, the silicon mold containing the resin in the liquid state was transferred in the curing oven (Figure 1d), and the curing cycle shown in Figure 2 was performed (4 h at room temperature + 2 h at 80 °C + 2 h at 120 °C).

After the manufacturing procedure of the samples (Figure 1b–d), the surfaces of the cured specimens were polished and then treated with spray paint to obtain a speckle suitable for the digital image correlation (DIC) software (Figure 1e). The curing cycle was defined starting from the technical datasheet of the cardanol-based epoxy systems provided by the supplier [27,28] and then modified considering similar works in the literature [17]. Moreover, Di Matteo et al. [32] performed a kinetic study on the curing ramp (Figure 2), considering the same epoxy systems used in this work. This study demonstrated that the cross-linking reaction was completed, and the resin system was fully cured after this curing cycle.

### 2.3. DSC Analysis

DSC experiments were performed for cured samples using DSC Q200 TA Instruments (New Castle, DE, USA) and aluminum sample pans. The samples were heated from −40 °C to 200 °C with a constant heating rate of 20 °C/min in a nitrogen (N_2_) atmosphere with a flow rate of 50 mL/min. DSC analysis was performed on all cured blends to assess the effect of the total bio-content increment on the T_g_ values and verify the absence of exothermic peaks after the curing process. The first heating scan aimed to erase the thermal history of the sample; T_g_ values were identified using the tangent intersection method between the initial and final temperatures at which glass transition takes place, from the second heating scan. A dedicated tool in the Universal Analysis software (TA Instruments, New Castle, DE, USA) was used.

### 2.4. Tensile Tests

Tensile tests were performed using an MTS Landmark (Eden Prairie, MN, USA) testing machine equipped with a 25 kN load cell, provided by the laboratories of the Material Technical Expertise department of Centro Ricerche Fiat (CRF, Stellantis, Turin, Italy). The testing machine was also equipped with a DIC system (LaVision GmbH, Göttingen, Germany) with DaVis 10 processing software (LaVision GmbH, Göttingen, Germany) for the acquisition of the strain distribution. Tensile tests were performed in quasi-static conditions (0.1 mm/s). A random speckle (Figure 1e) was sprayed on the specimen surface to track surface displacements and calculate material deformation by means of the DIC software. In particular, the tensile strain was computed during DIC data processing using one virtual strain gauge (6 mm × 10 mm) centered in the failure zone. The actual widths and thicknesses of each specimen were measured before the test in agreement with the standard ASTM D638 [31]. Three specimens were tested for each material. The modulus and strength values were computed considering the mean value of the three specimens.

## 3. Results and Discussions

### 3.1. DSC Analysis

**Figure 3:**
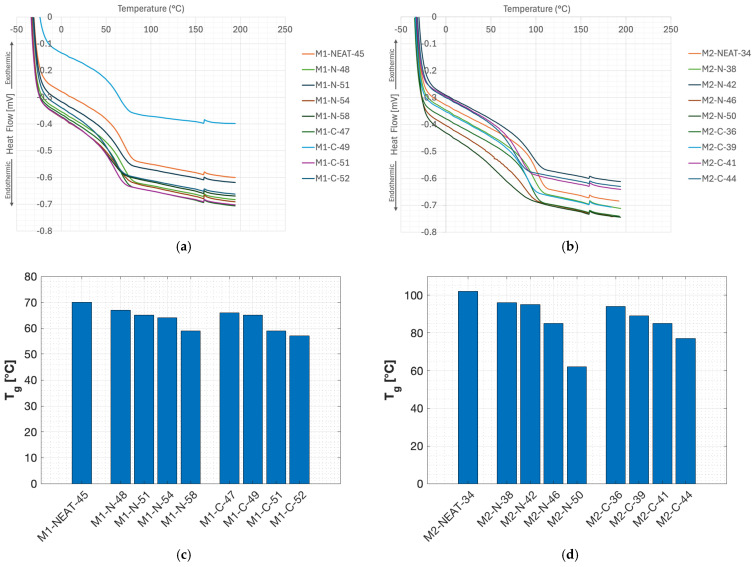
DSC curves for (**a**) M1 epoxy blends and (**b**) M2 epoxy blends and T_g_ values for (**c**) M1 epoxy blends and (**d**) M2 epoxy blends.

The DSC curves and T_g_ values measured by DSC analysis of the neat M1 and M2 epoxy systems considering different loadings of NC-514 (C) or NC-547 (N) are shown in Figure 3. All samples exhibited a single T_g_, indicating no phase separation.

The T_g_ of the systems cured with the 2002B hardener (Figure 3c) was significantly lower than that of the systems cured with the 2401B hardener (Figure 3d). In particular, the M1-NEAT-45 resin showed a T_g_ of 70 °C, which is comparable to the measurement obtained by Mu and Vaughan in their work [17] (67 °C). A decrease in T_g_ was observed with increasing amounts of N or C. Moreover, in the case of M1 epoxy blends (Figure 3c), the T_g_ decreased from 70 °C (M1-NEAT-45) to 57 °C (19% reduction) upon incorporating 40 wt.% of C (M1-C-52). Meanwhile, the decrease in T_g_ after adding 40 wt.% of N (M1-N-58) was up to 59 °C (16% reduction) compared to the neat formulation. The M2-NEAT-34 resin showed a T_g_ of 102 °C, which is comparable to the measurement obtained by Mu and Vaughan in their work [17] (97 °C). As for the M1 epoxy blends (Figure 3c), a decrease in T_g_ with increasing amounts of N or C was observed also for M2. In the case of M2 epoxy blends (Figure 3d), the T_g_ decreased from 102 °C (M2-NEAT-34) to 77 °C (24% reduction) upon incorporating 40 wt.% of C (M2-C-44), whereas the decrease in T_g_ by adding 40 wt.% of N to the neat formulation (M2-N-50) was up to 62 °C (39% reduction). This decrease in T_g_ could be attributed to flexibility granted by N and C [20,23]. In particular, C is characterized by a flexible chain situated in the backbone of its chemical structure, whereas N contains flexible groups in the side chain of the backbone and might induce less flexibility in the epoxy blends. Further, N has a greater number of epoxide groups; thus, it can lead to more cross-linking during the curing reaction, resulting in the overall decrease in flexibility in epoxy blends made with N [20]. Overall, the T_g_ values in this study decreased constantly by increasing the amount of C and N in both the M1 (Figure 3c) and M2 (Figure 3d) formulations, hence increasing the total bio-content of the final epoxy resin blends.

### 3.2. Tensile Tests

**Figure 4:**
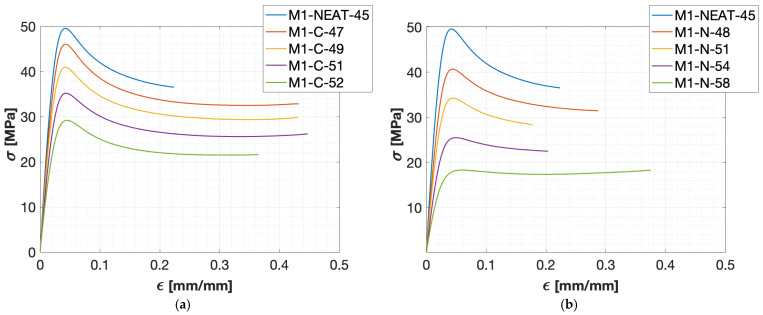

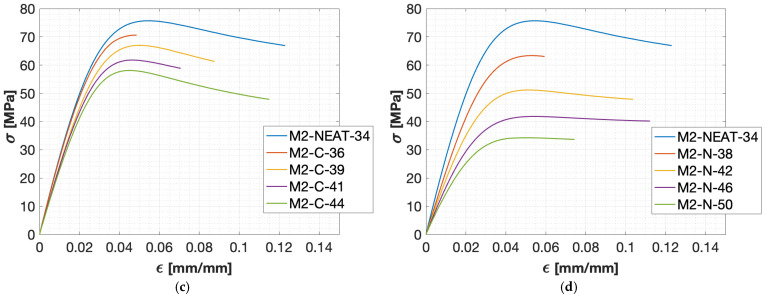
Engineering stress–strain curves at increasing bio-contents: (**a**) M1-C; (**b**) M1-N; (**c**) M2-C; and (**d**) M2-N.

Figure 4 shows the mean experimental engineering stress–strain curves for the epoxy resin blends considered in this study.

**Figure 5:**
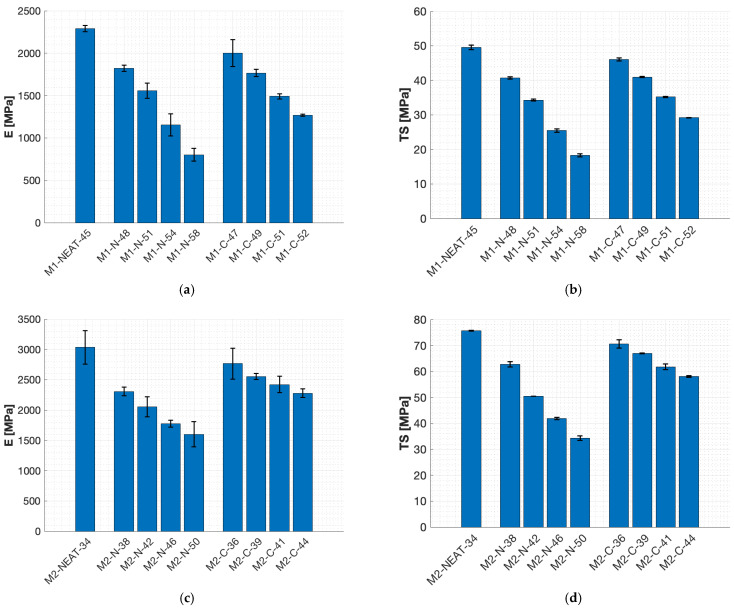
Experimental tensile test results for the considered epoxy blends: (**a**) tensile modulus for the M1 blends; (**b**) tensile strength for the M1 blends; (**c**) tensile modulus for the M2 blends; and (**d**) tensile strength for the M2 blends.

Overall, the curves obtained for the tensile tests of the M1-C blends (Figure 4a) showed lower peak values and a gradual reduction in the strain-softening effect as the total bio-content of the blends increased (Table 2). This phenomenon is even more relevant considering the addition of N (M1-N, Figure 4b), probably due to the higher flexibility effect of N [17,21] compared to C. Indeed, the neat M1-NEAT-45 epoxy system (Figure 4a,b) showed a pure post-yielding strain-softening behavior and an almost-pure post-yielding strain-neutral behavior for M1-N-58 (Figure 4b). Moreover, the tensile curves for the M1-C blends (Figure 4a) showed a post-yielding strain-softening behavior followed by an almost strain-neutral behavior at higher strains (>0.25). Overall, the M1-C epoxy blends generally showed higher strain values at failure. The curves obtained from the tensile tests of the M2 epoxy blends (Figure 4c,d) showed different tensile behaviors than those obtained from M1 (Figure 4a,b). Indeed, the tensile behavior showed post-yielding strain-softening for all M2-C blends (Figure 4c). In contrast, the M2-N blends, up to M2-N-42 (Figure 4d), showed a pure post-yielding strain-softening behavior, and, for higher loadings of N, the curves showed an almost-pure strain-neutral behavior (M2-N-46 and M2-N-50). Overall, the M2 blends could reach higher peak values than the M1 blends, considering the same loading of C (Figure 4a,c) and N (Figure 4b,d). This behavior could be justified by considering the starting mechanical properties of the M2-NEAT-34 epoxy system, which were higher compared to M1-NEAT-45 (Table 1). Moreover, the blends obtained using the M1 epoxy system (Figure 4a,b) showed strain at failure values much higher than those obtained using M2 (Figure 4c,d). Due to the incorporation of C and N in the neat compositions, a significant decrement in the tensile modulus (E) and tensile strength (TS) was observed. Figure 5 shows the variability in E and TS compared to the total bio-content of the blends for both M1 and M2.

The tensile moduli obtained for the neat formulations were 2.29 GPa (M1-NEAT-45) and 3.03 GPa (M2-NEAT-34). These values were in agreement with those provided by the resin supplier (Table 1) and comparable with (or even better than) conventional non-bio-based epoxy resins [6] and the most-used bio-based epoxy resins with similar bio-contents [6,8]. The blends obtained by adding 40 wt.% of C showed tensile modulus reductions from the values for the corresponding neat formulations (M1-NEAT-45 and M2-NEAT-34) of about 45% and 25% for M1-C-52 and M2-C-44, respectively. The reduction was even more significant for epoxy blends containing N. Indeed, the modulus decreased from 2.29 GPa (M1-NEAT-45) to 0.80 GPa for M1-N-58 (65% reduction). A reduction of about 47% was observed, passing from 3.03 GPa (M2-NEAT-34) to 1.60 GPa (M2-N-50). A similar decreasing behavior was also observed for the TS values. This detrimental effect on the mechanical behavior of the bio-based epoxy blends could be attributed to the flexible chains present in the backbone of C and N [21]. Indeed, C has a branched structure with flexible moiety in the main chain, whereas N has a linear structure with flexible C_15_H_27_ side chains [20]. These flexible structures can influence the toughening characteristics of the resultant epoxy blends. Moreover, considering the incorporation of 40 wt.% of N or C, the total drop from the neat system in terms of E and TS values was higher for the M1 (Figure 5a,b) than M2 blends (Figure 5c,d). Indeed, considering the M1-NEAT-45 material, the total drop in E was around 65% and 45% for M1-N-58 and M1-C-52, respectively. Similarly, the total drop in TS was around 63% and 41% for M1-N-58 and M1-C-52, respectively. On the other hand, considering the M2-N-50 material, the total drop in E and TS from the neat formulation was around 47% and 55%, respectively. Similarly, the total drop in E and TS from the neat formulation was around 25% and 23%, respectively, considering M2-C-44.

Epoxy blends obtained by adding the novolac-based resin N showed lower mechanical properties (E and TS) compared to the blends obtained using C resin, considering the same weight loading and neat epoxy system. For example, the E values for the blends obtained by adding 40 wt.% of N (M1-N-58 or M2-N-50) were smaller, in the range of 30–37%, with respect to those obtained by adding 40 wt.% of C (M1-C-52 or M2-C-44). The smaller the amount of N or C incorporated into the epoxy formulation, the less significant this difference was. Indeed, the E values for the blends obtained by adding 20 wt.% of N (M1-N-51 or M2-N-42) were smaller, in the range of 13–20%, with respect to those obtained by incorporating 20 wt.% of C (M1-C-49 or M2-C-39). Considering both the M1 and M2 blends, a linear decreasing trend in E and TS was observed by increasing the total bio-content of the epoxy resin blends.

In Table 4, some of the most common bio-based, partially bio-based, and non-bio-based epoxy systems present in the literature and on the market are reported, sorted from the highest bio-content to the lowest, as specified in the technical data sheets (TDSs).

**Figure 6:**
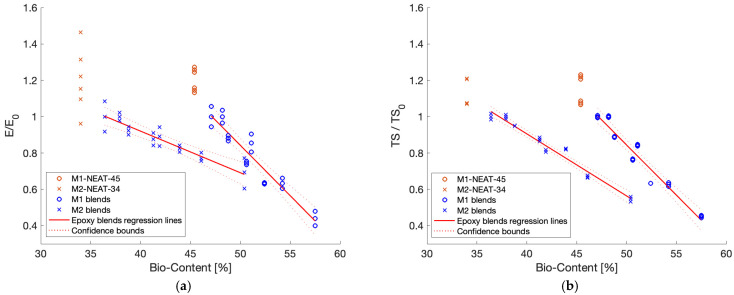
Normalized values of (**a**) E and (**b**) TS on the first resin blend for each epoxy mixture.

Cardolite products can reach very high bio-contents since both parts of the epoxy system (resin + hardener) are bio-based products. Indeed, the M1-N and M2-C blends showed a good compromise between mechanical properties, T_g_ values, and total bio-content of the final formulations. The M1-N blends were characterized by a very high bio-content (>45.4%), T_g_ values all over 60 °C, and mechanical properties (E and TS) comparable to the most common commercial epoxy systems characterized by similar or even lower bio-contents (Table 4). In comparison, the M2-C blends were characterized by bio-contents (>34%) higher than most epoxy systems on the market (Table 4), T_g_ values from 80 °C to 100 °C, and mechanical properties (E and TS) comparable to the most common commercial epoxy systems used for structural applications characterized by similar or lower bio-contents (Table 4). In particular, M2-C-39 had a bio-content of 39%, and its modulus (E = 2.55 GPa, Figure 5a) and strength (TS = 67 MPa, Figure 5b) were, respectively, 27% and 6% lower than the corresponding Sicomin^®^ product with a comparable total bio-content [35], but the T_g_ value of M2-C-39 was 10% higher (89 °C, Figure 3d), making this material more suitable for automotive applications. Moreover, the neat formulations considered in this paper (M1-NEAT-45 and M2-NEAT-34) showed comparable properties to most petrol-based epoxy resin systems on the market, making these commercial products suitable for reducing the environmental impact of final composite materials, with minimal alteration of the final products and their manufacturing process. The decrease in E and TS relative to the bio-content (Figure 6) was analyzed by considering the dispersion of all experimental data for the two neat epoxy systems, M1 and M2. The values for E/E_0_ and TS/TS_0_ were computed to compare the variation in the experimental data. These values were calculated by dividing the value of the considered material by the value of the first blend produced using the same epoxy system. For example, all the values for the M1-C blends (neat formulations included) were divided by the value of M1-C-47, for both E (Figure 6a) and TS (Figure 6b) values.

It is interesting to note that the regression lines for both the experimental E and TS data obtained for the M1 blends decreased following the same linear model (y_M1_ = −0.055x + 3.6). The dispersion of the data was acceptable for both the M1 and M2 blends. The M2 blends were less sensitive to mechanical property deterioration after adding C and N resins to the base formulation.

**Figure 7:**
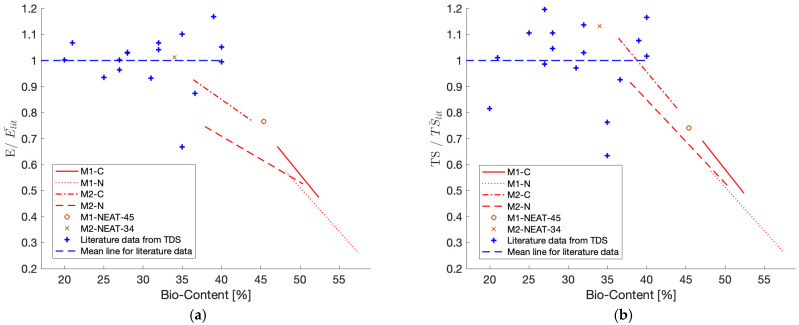
Normalized values on the mean of all data in the literature (Table 4) considering (**a**) moduli and (**b**) strengths.

Moreover, to make a comparison with the data present in the literature (Table 4), a further normalization is shown in Figure 7, dividing all the data by the average values of the literature moduli (${\bar{E}}_{lit}$) and strengths (${\bar{TS}}_{lit}$). More precisely, ${\bar{E}}_{lit}$ and ${\bar{TS}}_{lit}$ are the average values computed on all the moduli and strengths (respectively) reported in Table 4. The regression lines for the M1 and M2 blends, shown in Figure 6, are multiplied by E_0_/${\bar{E}}_{lit}$ (or TS_0_/${\bar{TS}}_{lit}$) to obtain the same normalization E/${\bar{E}}_{lit}$ and compare the trends of both experimental and literature data to the same mean modulus (${\bar{E}}_{lit}$) or strength (${\bar{TS}}_{lit}$) value.

Most products in the literature (Table 4) show properties above the mean value, but their bio-content does not exceed 40%. In particular, M2-NEAT-34 shows an E value comparable to ${\bar{E}}_{lit}$ and, in the case of TS (Figure 7b), the value is much higher than ${\bar{UTS}}_{lit}$. Among all the materials investigated in this work, the M2-C blends show the closest values to the average presented in the literature. In addition, they show bio-contents and T_g_ values higher than most of the data available in the literature, which is favorable for adopting these resins.

## 4. Conclusions

In the present paper, the effect of the increase in the total bio-content on the tensile properties of resin blends obtained by mixing commercially available cardanol-based epoxy resins and hardeners was assessed. Different total bio-contents were obtained starting from two different cardanol-based neat epoxy systems taken as a reference (M1-NEAT-45 and M2-NEAT-34) by adding different weight loadings of two high-bio-content resins: NC-514 (C) and NC-547 (N). The following conclusions could be drawn:

- A reduction in the Tg, E, and TS values was observed by increasing the total bio-content. The decrease in the properties highlighted an increase in flexibility when increasing the content of N and C.
- The increase in the total amount of bio-content led to a linear reduction in the E and TS values (Figure 6 and Figure 7).
- The stress–strain curves obtained through the tensile test (Figure 4) exhibited lower peak values and a reduction in the strain-softening effect as the total bio-content of the blends increased (Table 2). This phenomenon was more relevant considering the addition of N resin as opposed to C.
- The neat cardanol-based epoxy systems had comparable properties to the petrol-based epoxy resins commonly used for structural applications, and they were characterized by bio-contents higher than most epoxy systems on the market (Table 4).

In conclusion, the replacement of petrol-based epoxy resins with bio-based ones would reduce the environmental impact [11] and carbon footprint [10,16] of final composite materials with minimal alterations in the final product and its manufacturing process.

## Figures and Tables

**Table 1:** Chemical and mechanical properties of the two cardanol-based neat epoxy systems [27,28].

	Epoxy System 2501A + 2401B	Epoxy System 2501A + 2002B
Mixing ratio by weight	100:31	100:52
Mix viscosity @ 25 °C [cPs]	905	1100
Pot life ^1^, 100 g mix @ 23 °C [min]	95	58
Peak exotherm, 100 g mix @ 23 °C [°C]	30	36
Ultimate glass transition temperature ^2^ [°C]	100	73
Tensile strength [MPa]	69	52
Tensile modulus [MPa]	3134	2599
Tensile elongation at break [%]	6.6	11.3
Bio-based content [% wt.]	34.0%	45.4%

^1^ Pot life is measured when the formulation reaches a limit viscosity of 10,000 cPs starting from the reference temperature. ^2^ DSC scan from 0 to 200 °C at 20 °C/min, 2nd run.

**Table 2:** Stochiometric parts by weight of the M1 resin blends and total bio-content.

Nomenclature	Resin 2501A [g]	Resin NC-547 [g]	Resin NC-514 [g]	EEW (Resin Blend) [g/eq.]	PHR 2002B [g]	Total Bio-Content
M1_NEAT_45	100	-	-	200.00	52.00	45.4%
M1_N_48	90	10	-	216.49	48.04	48.2%
M1_N_51	80	20	-	235.96	44.08	51.1%
M1_N_54	70	30	-	259.26	40.11	54.2%
M1_N_58	60	40	-	287.67	36.15	57.5%
M1_C_47	90	-	10	211.18	49.25	47.1%
M1_C_49	80	-	20	223.68	46.49	48.8%
M1_C_51	70	-	30	237.76	43.74	50.6%
M1_C_52	60	-	40	253.73	40.99	52.4%

**Table 3:** Stochiometric parts by weight of the M2 resin blends and total bio-content.

Nomenclature	Resin 2501A [g]	Resin NC-547 [g]	Resin NC-514 [g]	EEW (Resin Blend) [g/eq.]	PHR 2401B [g]	Total Bio-Content
M2_NEAT_34	100	-	-	200.00	31.00	34.0%
M2_N_38	90	10	-	216.49	28.64	37.9%
M2_N_42	80	20	-	235.96	26.28	41.9%
M2_N_46	70	30	-	259.26	23.91	46.1%
M2_N_50	60	40	-	287.67	21.55	50.4%
M2_C_36	90	-	10	211.18	29.36	36.4%
M2_C_39	80	-	20	223.68	27.72	38.8%
M2_C_41	70	-	30	237.76	26.08	41.3%
M2_C_44	60	-	40	253.73	24.44	43.9%

**Table 4:** Summary of epoxy resin systems’ mechanical properties, T_g_ values, and total bio-content, as specified in TDSs.

Supplier	Name	Bio-Based?	Name	Bio-Based?	E	TS	T_g_	Bio-Content	Reference
	Epoxy Resin		Curing Agent		[GPa]	[MPa]	[°C]	[%]	
Cardolite Corp. (Gent, Belgium)	FormuLite 2501A	yes	FormuLite 2002B	yes	2.6	52	73	45.4	this work [27]
Sicomin (Pluguffan, France)	Greenpoxy 56	yes	SD7561	no	2.98	68	78	40	[33]
Sicomin (Pluguffan, France)	Greenpoxy 56	yes	SZ 8525	no	3.15	78	87	40	[34]
Sicomin (Pluguffan, France)	Greenpoxy 56	yes	SD 4772	no	3.5	72	80	39	[35]
Cardolite Corp. (Gent, Belgium)	FormuLite 2500A	yes	Formulite 2401B	yes	2.615	62.0	92.0	36.6	[36]
Sicomin (Pluguffan, France)	Greenpoxy 56	yes	SD Surf Clear	no	3.30	51	78	35	[37]
Gurit (Bristol, TN, USA)	AMPRO BIO	yes	AMPRO Slow	yes	2	42.4	49	35	[38]
Cardolite Corp. (Gent, Belgium)	FormuLite 2501A	yes	Formulite 2401B	yes	3.13	69	100	34	this work [28]
Entropy Resins (Bay, MI, USA)	305	yes	CPS (slow)	yes	3.2	68.9	68.0	32	[39]
Entropy Resins (Bay, MI, USA)	QC resin	yes	Slow hardener	yes	3.12	76	82	32	[40]
Easy Composites Ltd. (Stoke on Trent, UK)	IB2 resin	yes	IB2 hardener	no	2.79	65	85	31	[41]
Sicomin (Pluguffan, France)	Infugreen 810	yes	SD 8822	no	3.09	74	85	28.0	[42]
Sicomin (Pluguffan, France)	Infugreen 810	yes	SD 4775	no	3.08	70	79	28	[43]
Cardolite Corp. (Gent, Belgium)	FormuLite 2502A	yes	FormuLite 2401B	yes	2.89	66.0	88.0	27.0	[44]
Sicomin (Pluguffan, France)	Greenpoxy 33	yes	SZ 8525	no	3	80	120	27	[45]
Sicomin (Pluguffan, France)	Greenpoxy 33	yes	SD 4771	no	2.8	74	100	25	[46]
Entropy Resins (Bay, MI, USA)	CLR	yes	CLS (slow)	yes	3.2	67.6	61	21	[47]
Entropy Resins (Bay, MI, USA)	BRT	yes	CLS (slow)	yes	3.2	67.6	57	21	[48]
Entropy Resins (Bay, MI, USA)	CCR	yes	CCS (slow)	yes	3	54.5	52	20	[49]
Huntsman (The Woodlands, TX, USA)	Araldite LY 556	no	Aradur 917	no	3.3	93	155	//	[50]
Easy Composites Ltd. (Stoke on Trent, UK)	IN2	no	AT30 Slow	no	3.00	68.5	88	//	[51]
Sicomin (Pluguffan, France)	SR8100	no	SD8822	no	2.85	71	85	//	[52]

## Data Availability

The data presented in this study are available upon request from the corresponding author. The data are not publicly available due to privacy or ethical restrictions.
